# Mobile phone brief intervention applications for risky alcohol use among university students: a randomized controlled study

**DOI:** 10.1186/1940-0640-9-11

**Published:** 2014-07-02

**Authors:** Mikael Gajecki, Anne H Berman, Kristina Sinadinovic, Ingvar Rosendahl, Claes Andersson

**Affiliations:** 1 Karolinska Institutet, Department of Clinical Neuroscience, Center for Psychiatric Research, Stockholm, Sweden; 2 Stockholm Center for Dependency Disorders, Stockholm, Sweden; 3 Department of Criminology, Malmö University, Malmö, Sweden

**Keywords:** Randomized controlled trial, Problem drinking, Alcohol abuse, College, University, Smartphone, Mobile phone, eHealth, mHealth, Brief intervention

## Abstract

**Background:**

Brief interventions via the internet have been shown to reduce university students’ alcohol intake. This study tested two smartphone applications (apps) targeting drinking choices on party occasions, with the goal of reducing problematic alcohol intake among Swedish university students.

**Methods:**

Students were recruited via e-mails sent to student union members at two universities. Those who gave informed consent, had a smartphone, and showed risky alcohol consumption according to the Alcohol Use Disorders Identification Test (AUDIT) were randomized into three groups. Group 1 had access to the Swedish government alcohol monopoly’s app, Promillekoll, offering real-time estimated blood alcohol concentration (eBAC) calculation; Group 2 had access to a web-based app, PartyPlanner, developed by the research group, offering real-time eBAC calculation with planning and follow-up functions; and Group 3 participants were controls. Follow-up was conducted at 7 weeks.

**Results:**

Among 28574 students offered participation, 4823 agreed to join; 415 were excluded due to incomplete data, and 1932 fulfilled eligibility criteria for randomization. Attrition was 22.7–39.3 percent, higher among heavier drinkers and highest in Group 2. Self-reported app use was higher in Group 1 (74%) compared to Group 2 (41%). Per-protocol analyses revealed only one significant time-by-group interaction, where Group 1 participants increased the frequency of their drinking occasions compared to controls (p = 0.001). Secondary analyses by gender showed a significant difference among men in Group 1 for frequency of drinking occasions per week (p = 0.001), but not among women. Among all participants, 29 percent showed high-risk drinking, over the recommended weekly drinking levels of 9 (women) and 14 (men) standard glasses.

**Conclusions:**

Smartphone apps can make brief interventions available to large numbers of university students. The apps studied using eBAC calculation did not, however, seem to affect alcohol consumption among university students and one app may have led to a negative effect among men. Future research should: 1) explore ways to increase user retention, 2) include apps facilitating technical manipulation for evaluation of added components, 3) explore the effects of adapting app content to possible gender differences, and 4) offer additional interventions to high-risk users.

**Trial registration:**

clinicaltrials.gov: NCT01958398.

## Introduction

Approximately 37–50 percent of college and university students consume alcohol at risky levels [1,2]. Several effective methods for reducing risky and hazardous drinking among students have been identified, using information and early intervention as well as screening and brief intervention (SBI) [3-5]. One central focus of these methods concerns an individual’s *intentions* to drink and his or her behavioral *control* over alcohol consumption. The Theory of Planned Behavior (TPB) [6] proposes that a person’s intentions are the foremost determinants of whether the behavior is performed or not. Information about the behavior that is available at any given moment influences intentions as well as actions. A number of studies confirm that drinking intentions among college students are predictive of drinking behaviors, and that the intended degree of drinking influences the level of the actual drinking [7-9]. A second central focus of effective methods is the use of *protective cognitive and behavioral strategies* that aim to limit alcohol consumption and collateral consequences [10]. Interventions that include skill training for protective behavioral strategies have shown an association with less alcohol use and alcohol-related consequences [11-13]. Many alcohol-related prevention programs tailored to college students include a skills training component aimed at reducing intoxication when drinking [4,14,15].

### Technology and brief intervention

Students and young people often are reluctant to seek interventions for reducing their drinking behavior when such interventions are provided by health care professionals [16]. Short, technology-based interventions for younger people might contribute to the availability and access to interventions that could increase health-oriented behavior change. Indeed, the beneficial effects of technology-based brief interventions for problematic alcohol use have been shown for student populations in several reviews and meta-analyses, where the interventions were delivered via computer—with and without internet access [17-21].

Since the advent of the smartphone, i.e., mobile telephones providing advanced functionality in addition to that of regular telephony, more and more people have immediate access to fairly powerful computers close at hand. In 2013, 94 percent of individuals between 16 and 25 years of age had access to a smartphone and 88 percent of those 26–35 years old had such access [22]. Smartphones make it possible to download and run software applications, commonly referred to as “apps,” the use of which has exploded since 2008 when the two major venues for downloading apps opened—Apple’s App store and Google’s Play (formerly Android market).

One app sector targets health-related behaviors such as smoking and obesity, including problematic alcohol consumption. Although over 3000 apps focus on alcohol consumption, recent reviews indicate that many of the apps are intended to *encourage* drinking; and while apps offering support in *reducing* problematic alcohol use do exist, very little research evaluating their effects has been published [23,24].

### Estimating blood alcohol content

One of the most prevalent components of smartphone apps related to alcohol consumption is the functionality of calculating and displaying an individual’s estimated blood alcohol concentration (eBAC) [23,24]. Learning to calculate the eBAC and relating it to its effects on the individual—both in terms of desirable and positive effects as well as harmful and negative effects—is an integral part of the Alcohol Skills Training Program (ASTP) [25] and Brief Alcohol Screening and Intervention for College Students Program (BASICS) [26], information and early intervention methods that have good documented support [3,4,27]. Calculating and displaying an individual’s eBAC is a form of personalized feedback, and personalized feedback via mail or computer has been found to be an effective brief intervention [28].

The background motivations for conducting the present study were twofold. First, the Swedish government-owned alcohol monopoly (*Systembolaget*), which has an explicit mandate to limit health-related harm caused by alcohol, launched its own app, *Promillekoll,* in late 2012, offering users real-time feedback in the form of eBAC. The stated purpose of the app was to reduce risky and harmful alcohol drinking among university students, but its effects have hitherto not been studied scientifically. Second, the senior author of this article had conducted a study on an automated telephony and web-based intervention that offered university students eBAC calculation to reduce risky drinking [29]. Our research group adapted this intervention into an app format that included an added planning and follow-up component under the name *PartyPlanner.*

## Aims

This study investigates the effects of two Swedish-language smartphone apps with real-time eBAC calculation and feedback among university students with established levels of risky drinking. Each app was compared to assessment-only controls. We hypothesized that using each of these apps would lead to greater reductions in risky drinking than those seen in the assessment-only control group. Given the differing levels of alcohol consumption between men and women, we conducted a secondary analysis to explore whether there were any gender differences for these two apps in terms of alcohol outcomes. Earlier studies on gender effects for SBI outcomes are somewhat inconclusive, with some studies reporting gender differences [30,31] but later research showing no such differences [32]. As far as we know, gender differences in the effects of smartphone-delivered SBIs have not been previously studied.

In summary, we report analyses of outcomes between groups with access to each app in comparison to a control group, additionally examining possible gender differences. We further discuss the implications of these results for university students as well as future research recommendations.

## Methods

### Participants

The student unions at Stockholm University and the Royal Institute of Technology in Stockholm, Sweden, provided our research group with e-mail addresses for their current members. We e-mailed study information and a web page link to all addresses on the lists provided. Potential participants were informed that completing baseline and follow-up questionnaires in the study would automatically include them in a lottery with three iPad devices as prizes. Those interested in participation clicked a link directing them to a web page where they received further information on the Swedish Personal Data Act, and where they could indicate their consent to participate in the study. Individuals giving informed consent participated in a data intake process requiring registration of their mobile phone number, gender, age, and weight. Participants were also asked whether they had access to a smartphone running either of the two operating systems, iOS or Android. Thereafter, participants filled out baseline questionnaires consisting of the Daily Drinking Questionnaire (DDQ) [33] and the Alcohol Use Disorders Identification Test (AUDIT) [34]. Participants with an AUDIT score indicating at least hazardous consumption (≥6 for women and ≥ 8 for men) [35] and having a smartphone running either iOS or Android were randomized to one of the two application conditions or to an assessment-only control group. Those not fulfilling these criteria were excluded from randomization. One reminder e-mail was sent out 2 days after the first e-mail to those who had not responded. Study registration was open for one week. Participants were informed that some students would be contacted with an e-mail containing a link to a smartphone app, and that all would be asked to fill in follow-up questionnaires 6 weeks after registration, a timeframe based on a study previously conducted by the research group and commonly used in student alcohol studies [29]. No feedback on baseline consumption levels was given to any of the participants, regardless of group assignment.

### Randomization

All eligible participants were randomized to one of the two interventions or to a control group with the ratio (1:1:1), using the randomization function in the IBM SPSS Statistics for MacOS X, Version 19 (IBM Corp, Armonk, NY, USA). Participants randomized to an intervention were sent an e-mail 10 days after the first information e-mail was sent (3 days after study registration ended) with a link to access the app to which they had been randomized. Participants in the intervention groups were instructed to use the app during the following weeks, in association with events where alcohol would be consumed. There were no further prompts to use the application during the period leading up to the follow-up. Participants were not blind as to whether or not they were allocated to an intervention condition, but they were not informed that one of the interventions included a planning and follow-up component. The randomization process was fully automated.

### Interventions

1: *Promillekoll app (tr. “Check your BAC”):* As noted above, this app was developed by the Swedish government’s Systembolaget and is publicly downloadable for iPhone and Android smartphones. This app was released for public use on September 25, 2012. The user can register his/her alcohol consumption in real time, where the app displays the user's current eBAC. The Promillekoll app is theoretically based on the assumption that information about one’s own real-time eBAC levels can contribute to one’s protective cognitive and behavioral strategies. Promillekoll also offers a number of specific strategies to maintain alcohol consumption at a level that is not harmful—in this case, 0.06 percent BAC. A further mechanism, congruent with the Theory of Planned Behavior (TPB) [6], is that providing information and feedback on risky levels of eBAC modifies the intention to consume alcohol.

The application warns the user if the drink entered will result in an eBAC over 0.06 percent and only displays values up to 0.08 percent. It also provides information texts on alcohol and BAC. The study was conducted using a publicly available app, which is constructed as a stand-alone application that can be used offline. No user data are collected. The research group had no influence on the development or functionality of the app.

2: *PartyPlanner app.* In order to further develop and test the idea of modifying drinking intentions with an app, our research group developed a new app, “PartyPlanner,” with the functionality of simulating or planning a drinking event beforehand and then comparing the simulation to the real-time event afterwards. Our hypothesis is that setting up a plan for personal eBAC levels *before* the drinking event might explicitly modify the user’s drinking intentions by adapting user perceptions of risk to reality. The app user would then be able to pace his or her drinking based on a more realistic view of the amount of alcohol actually corresponding to a certain eBAC level. Comparing eBAC levels *after* the event could increase skillfulness in future protective behavioral strategies and increase control when drinking alcohol.

Thus, in addition to registering alcohol consumption with instant visual eBAC similar to Promillekoll, this app gives the user the opportunity to simulate an event where alcohol will be consumed ahead of time. The app displays the eBAC level at distinct time points throughout the drinking occasion, both for pre-party simulations and real-time registrations. Color codes indicate whether the eBAC is at a risky level. The real-time registration with feedback can be used as a standalone function; i.e., without having made a prior plan. However, if there is a plan, the user can visually compare the plan with the logged real-time event after the actual drinking occasion. In contrast to Promillekoll, the PartyPlanner app was launched as a so-called web app that requires internet connection using a web browser in order to facilitate additional development following this study and prior to possible future, wider, public accessibility. This app was developed by the authors in collaboration with Liquid Media AB.

### Control group

The third group was a control group that did not receive any intervention or feedback on risky drinking. Individuals allocated to this group did not receive any further information in the time between study registration and follow-up.

### Follow-up

Seven weeks after registration, all participants received an e-mail inviting them to answer the follow-up questionnaires. One reminder e-mail was sent 4 days after the first e-mail to initial nonresponders. The e-mails contained a link to an online questionnaire, where participants filled out the AUDIT, the DDQ, and answered questions on access to other interventions for reducing their alcohol use, such as speaking with someone else, using web-based services, or contacting professional treatment providers. Participants in the intervention groups were also asked whether they had actually used the app and how they liked it. The entire process was fully automated and no contact occurred with a human counterpart, but participants were provided with an e-mail address for technical support and questions regarding the study.

The follow-up e-mail was sent after 7 weeks rather than the originally planned 6 weeks due to unforeseen technical difficulties. The reason for the chosen timespan of 6 weeks was to facilitate comparison with an earlier study on digital interventions by the study group targeting the same population [29].

#### Seasonality

The study took place in March and April 2013. Swedish university programming is not based on the concepts of midterms or finals, so there were no uniform examination periods during this time. During the intervention period, two major public holidays occurred: Easter and Walpurgis Night (April 30). Walpurgis Night is traditionally connected to partying, with high alcohol consumption in Swedish culture.

### Ethics

**Figure 1:**
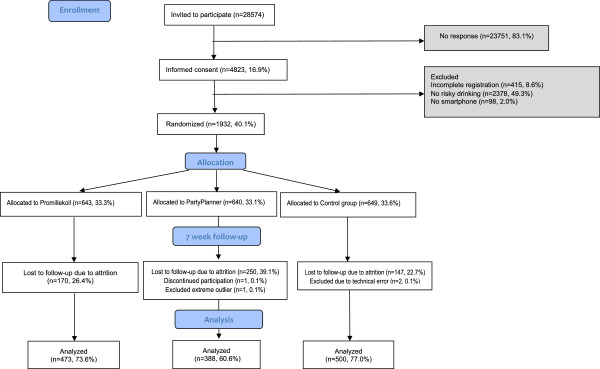
**Consort diagram of the trial.** Shaded areas were not included in analyses.

The study was approved by the regional ethics vetting board in Stockholm (ref. nr. 2012/1126–31/1). Since Swedish universities are not permitted to organize lotteries, the iPad lottery was conducted by the charity organization, Save the Children. See Figure 1 for a participant flowchart.

### Design

The study evaluated the effectiveness of access to one of two smartphone apps addressing risky alcohol use among university students in Stockholm, Sweden. A randomized, parallel, three-group, repeated-measures design was used in which alcohol-related outcomes of two smartphone intervention groups were separately compared to an assessment-only control group. Participants were assessed at baseline before trial and at follow-up 7 weeks later. The trial was registered at clinicaltrials.gov (ref. nr. NCT01958398).

### Measures

Participants’ alcohol consumption levels and BAC were investigated during the trial. To measure quantity and frequency of alcohol consumption, the DDQ [33] was used. The instrument was translated into Swedish by Malmö University, in collaboration with the University of Washington. Participants were asked to consider a typical week during the past month and state how many standard glasses of alcohol they drank and over how many hours for each day of the current week. They were also asked to report their peak alcohol consumption event during the past month in terms of how many standard glasses they drank during a self-reported number of hours. This measure has demonstrated good test-retest reliability in paper format [27].

The eBAC was calculated based on the values from the DDQ in conjunction with weight and gender for each individual. The formula used was the widely known Widmark formula, as modified and used by the United States National Highway Traffic Safety Administration: eBAC (in parts per mille, as is standard in Sweden) = ([number of standard glasses] × 12 grams)/([body weight in kg] × C) – [no. of hours] × 0.15), where C is a gender-specific constant of 0.68 for men and 0.55 for women [36]. In order to convert the eBAC to percentage values for this article, the values were divided by 10. In the study, a peak eBAC value was calculated as the eBAC of the peak alcohol consumption event of the past month. The mean eBAC value was calculated as the mean of the eBAC values specified for each day during the typical week reported in the DDQ.

The AUDIT [34] consists of 10 questions measuring consumption and signs of harm and dependence in relation to alcohol. The instrument was used to assess whether problematic drinking was present and, if so, its severity. The Swedish version has shown good internal consistency. The paper version has yielded Cronbach’s α values of 0.81–0.82 [35,37], and the internet version has yielded Cronbach’s α values of 0.80–0.93 [38,39].

In order to assess the app usage, self-reported data were gathered at follow-up on whether the apps had been used and, if so, how many times. Questions on the users’ perception of the apps were also asked at follow-up.

## Definitions

### Risky and problematic drinking

No level of alcohol drinking is known to be risk free, and there is no internationally agreed-upon amount defined as hazardous. Guidelines thus vary considerably, but risky use in terms of volume consumed is often defined either as a high weekly consumption or high consumption on one occasion (binge drinking). In Sweden, the Swedish National Institute of Public Health defines hazardous drinking for men as 14 or more standard glasses (in Sweden, 12 grams of pure alcohol) in a week, or five or more standard glasses per occasion. For women, the limits are nine or more standard glasses in a week or four or more standard glasses at any one occasion [40]. While binge drinking can be defined as four or five drinks per occasion, it can also be defined using BAC, where the level of 0.08 percent is commonly used [41]. In this study the slightly more conservative level of 0.06 percent was used, which is closer to the 0.055 percent BAC recommended in the BASICS program [26].

In this study, we defined problematic drinking based on an AUDIT score over the cutoff level for risky or hazardous drinking (≥6 for women and ≥ 8 for men), thus also including the categories harmful drinking (≥16 for men and women) and probable alcohol dependence (≥20 for men and women) [42].

### Statistical analyses

Descriptive statistics were used to describe baseline characteristics. Analysis of variance (ANOVA) was used to identify any baseline differences in age, AUDIT, mean eBAC, peak eBAC, quantity, frequency, and number of binge drinking occasions between the groups. Pearson’s chi-squared tests were used to determine differences between the groups in proportion of gender and the proportion of participants drinking more than the weekly recommendation. A linear mixed model analysis was used to identify changes over time in alcohol consumption outcomes: mean eBAC, peak eBAC, quantity, frequency, and number of binge drinking occasions. These analyses were conducted *per protocol*—that is, including only those participants who reported using the app they were assigned to—and controlling for three variables: the number of times the app was accessed, having spoken to someone about alcohol consumption during the past 12 months, and having accessed the publicly available Promillekoll app prior to the study. For comparison, *intention to treat* analyses were performed with all participants who were randomized to experimental groups and retaining baseline values for as many participants as possible. No data imputation procedures were applied.

Descriptive statistics, ANOVA, repeated-measures ANOVA, and Pearson’s chi-squared analyses were performed using IBM SPSS Statistics for MacOS X, Version 22 (IBM Corp, Armonk, NY, USA). Linear mixed model analyses were performed using Stata 13 (StataCorp. 2013. College Station, TX: StataCorp LP.). Values for averages and standard deviations are presented to three decimal places in order to make differences visually discernible.

### Exclusions

Two participants were identified as extreme outliers at baseline, one who entered a value of 70 standard glasses for the peak consumption occasion and another who indicated an age of 90 years. A third participant had left incomplete data at baseline, and was included in follow-up due to a technical problem. These three participants were excluded from all analyses.

## Results

### Participant characteristics

Baseline participant characteristics did not differ overall among participants randomized to the three arms of the trial (see Table 1). In total, the sample consisted of approximately equal numbers of men and women (48.3% male, 51.7% female). The mean AUDIT score (10.7, SD = 3.9) indicated hazardous drinking levels, with almost one-third (29.7%) of the participants drinking more on a weekly basis than the recommended Swedish guidelines of less than nine drinks for women and less than 14 for men per week.

**Table 1:** Baseline characteristics of students with risky alcohol use in a randomized brief intervention app trial

**Characteristic**	**Total (N = 1929)**	**PartyPlanner (n = 639)**	**Promillekoll (n = 643)**	**Control (n = 647)**	**p-values**^**a**^
Gender: M (%)/F (%)	931 (48.3)/998 (51.7)	296 (46.3)/343 (53.7)	334 (51.9)/309 (48.1)	301(46.5)/346 (53.5)	0.073
Age: mean (SD)	24.720 (4.809)	24.820 (4.631)	24.640 (4.991)	24.700 (4.804)	0.805
Measures of alcohol consumption: means (SD)					
AUDIT score (scale 0–40)	10.657 (3.896)	10.676 (3.944)	10.647 (3.718)	10.649 (4.026)	0.989
Quantity (standard glasses/week)	9.260 (6.282)	9.299 (6.439)	9.335 (6.236)	9.146 (6.178)	0.848
Frequency (drinking occasions/week)	2.270 (1.177)	2.337 (1.164)	2.185 (1.171)	2.288 (1.191)	0.063
Binge occasions (no. per week)	0.997 (0.855)	0.975 (0.851)	1.045 (0.861)	0.971 (0.852)	0.215
Average eBAC^b^ per week	0.017 (0.015)	0.018 (0.016)	0.017 (0.015)	0.017 (0.015)	0.386
Peak eBAC^c^ within past month	0.126 (0.803)	0.130 (0.825)	0.121 (0.077)	0.128 (0.081)	0.103
Percent (%) over weekly recommended level	29.7	31.1	30.8	27.2	0.230

^a^P-values are based on ANOVA for age, AUDIT, quantity, frequency, binge occasions, average eBAC, and peak eBAC; Pearson’s chi-square was used for gender and % over the weekly recommended level.

^b^Estimated average percentage BAC per week.

^c^Estimated average peak percentage BAC during the past month.

### Attrition

The overall attrition rate was 29.4 percent (n = 568); one participant who asked to terminate his study participation was included in the attrition group. An analysis comparing the attrition group with participants who completed the follow-up (“completers”) showed no significant differences in gender or age. However, participants in the attrition group had significantly higher scores on all outcome variables related to alcohol consumption, except for peak eBAC per month (see Table 2).

**Table 2:** Baseline characteristics for participants completing follow-up (“completers”) compared to participants who did not (“attrition group”)

	**Completers (n = 1361)**	**Attrition group (n = 568)**	**p-values**^**a**^
Gender: M (%)/F (%)	646(47.5)/715(52.5)	285(50.2)/283(49.8)	0.277
Age: mean (SD)	24.840 (4.931)	24.420 (4.493)	0.078
Measures of alcohol consumption: means (SD)			
AUDIT score (scale 0–40)	10.444 (3.716)	11.169 (4.257)	< 0.001
Quantity (standard glasses/week)	8.812 (5.696)	10.333 (7.398)	< 0.001
Frequency (drinking occasions/week)	2.213 (1.139)	2.405 (1.253)	0.002
Binge occasions (no. per week)	0.950 (0.807)	1.109 (0.951)	< 0.001
Average eBAC^b^ per week	0.161 (0.133)	0.198 (0.187)	< 0.001
Peak eBAC^c^ within past month	1.240 (0.760)	1.319 (0.897)	0.063
Percent (%) over weekly recommended levels	27.4	35.2	0.001

^a^P-values are based on student’s t-test for age, AUDIT, quantity, frequency, binge occasions, average eBAC, and peak eBAC; Pearson’s chi-square was used for gender and % over weekly recommended level.

^b^Estimated average percentage BAC per week.

^c^Estimated average peak percentage BAC during the past month.

Analyzed by group, attrition was not equal. The PartyPlanner group had a higher attrition rate, at 39.3 percent, compared to the Promillekoll (26.4%) and control (22.7%) groups [χ^2^ (2, 1929) = 46.633, p < 0.001]. A sub-analysis revealed that in the PartyPlanner group, all alcohol consumption-related baseline values were higher among the attrited individuals than among those who completed the follow-up; the same was true for all baseline values except for frequency and number of binge drinking occasions per week in the control group. For the Promillekoll group, there were no differences in baseline values between completers and the attrited group. Significantly more of the men (43.9%) in the PartyPlanner group did not complete follow-up in comparison to the women (35.3%) [χ^2^ (1, 639) = 4.975, p = 0.026]; there were no gender differences for follow-up rates in the other two groups.

### App use

Over one-third of the participants in all three groups had tried Promillekoll before initiation of the study. Fewer PartyPlanner participants (41.4%) reported having used the PartyPlanner app during the study period, compared to Promillekoll participants (74.1%; χ^2^ (1, 845) = 92.844, p < 0.001).

### Other interventions and prior experiences of Promillekoll

Between 0.4 percent and 0.6 percent of all study participants reported having used pharmaceutical medications in order to reduce alcohol consumption during the 12 months preceding the follow-up, and between 0.8 percent and 2.6 percent reported having accessed help other than medications or speaking to someone about their use.

Over 20 percent in each group reported at follow-up that they had spoken with someone about their alcohol consumption during the prior 12 months.

### Usability data

Users rated the two apps for ease of use, suitability, and likelihood of recommending it to a friend. The only significant difference between the two apps was that Promillekoll participants rated ease of use higher (4.0) than PartyPlanner participants (3.2) (see Table 3).

**Table 3:** Self-reported data on prior use of Promillekoll, speaking to someone about use, and app usability

**Measurement occasion**	**Questions**	**PartyPlanner (n = 155-7)**	**Promillekoll (n = 342)**	**Control group (n = 489)**	**Significance level (p-value)**
Baseline	Had you tried the Promillekoll app before agreeing to participate in this study? % of yes responses (n)	34.8 (54)	34.5 (118)	33.7 (165)	0.080
Follow-up	Have you spoken to someone regarding your alcohol consumption during the last 12 months? % of yes responses (n)	25.3 (39)	27.2 (93)	20.7 (101)	0.957
Follow-up	How did you experience using the app? Mean scores^a^ (SD) 1 = Very difficult 5 = Very easy	3.2 (1.1)	4.0 (1.1)	N/A	< 0.001
Follow-up	In your opinion, how suitable is an app of this kind for helping people with risky alcohol consumption? Mean scores^a^ (SD) 1 = Not at all 5 = Very suitable	3.6 (1.2)	3.4 (1.2)	N/A	0.171
Follow-up	Would you recommend using an app of this kind to a friend who wanted to keep track of his or her alcohol consumption? Mean scores^a^ (SD) 1 = Never 5 = Definitely	3.6 (1.2)	3.7 (1.3)	N/A	0.663

^a^Scores are given as discrete values on the scale of 1–5. The highest and lowest scores are defined in each cell under the question.

### Outcome analyses

Time-by-group interactions were investigated using linear mixed models analyses, separately comparing each app group (per protocol) to the control group and controlling for the number of app-use occasions, earlier use of Promillekoll, and having spoken to someone about alcohol consumption over the past 12 months. A Bonferroni correction of p = 0.003 was applied throughout. For the PartyPlanner group, no significant time-by-group interactions for any outcome measures occurred. For the Promillekoll group, app users showed a significant increase in drinking frequency compared to the control group [Z = 3.39, p = 0.001] (see Table 4).

**Table 4:** **Baseline and 7-week follow-up alcohol consumption outcomes, comparing intervention groups to controls**^**a**^

**Measures of alcohol consumption in means (SD)**	**Control**		**PartyPlanner (n = 153)**		**PartyPlanner compared to Control**^**a **^**linear mixed models**	**Promillekoll (n = 341)**		**Promillekoll compared to Control**^**a **^**linear mixed models**
	**Baseline (n = 489)**	**Follow-up (n = 489)**	**Baseline**	**Follow-up**	**Time x Group p-value**	**Baseline**	**Follow-up**	**Time x Group p-value**
Quantity (standard glasses/week)	9.146 (6.178)	8.619 (6.281)	8.570 (6.122)	8.317 (6.449)	0.816	9.617 (6.263)	9.746 (7.053)	0.411
Frequency (drinking occasions/week)	2.288 (1.191)	2.152 (1.192)	2.165 (1.122)	2.171 (1.232)	0.225	2.242 (1.196)	2.362 (1.234)	< 0.001
Binge occasions (no per week)	0.971 (0.852)	0.897 (0.838)	0.880 (0.760)	0.791 (0.822)	0.507	1.082 (0.875)	1.018 (0.837)	0.629
eBAC^b^/week	0.017 (0.015)	0.016 (0.015)	0.016 (0.013)	0.015 (0.016)	0.914	0.017 (0.014)	0.017 (0.018)	0.750
Peak eBAC^c^/month	0.128 (0.082)	0.118 (0.080)	0.126 (0.087)	0.122 (0.096)	0.649	0.108 (0.087)	0.119 (0.084)	0.776

^a^Analyses comparing the two intervention groups to controls included only individuals who reported actually using the assigned app. Analyses were controlled for the number of times participants reported using the app, previous use of the publicly available Promillekoll app, and self-reports on having spoken to someone in the past 12 months about their alcohol use.

^b^Estimated average percentage BAC for the week.

^c^Estimated average peak percentage BAC during the past month.

The three covariates were all significant: number of app-use occasions [Z = 8.24, p < 0.001]; having spoken to someone about alcohol consumption in the past 12 months [Z = 3.73, p < 0.001]; and having tried Promillekoll before the study [Z = 2.62, p = 0.001].

An intention-to-treat analysis including all users regardless of reported app use, and not controlling for any other factors, did not yield any significant differences between the groups.

At follow-up, the proportion of group members having a weekly consumption over the recommended level remained more than 25 percent (Control group: 26.4%, PartyPlanner: 26.7%, and Promillekoll: 28.8%).

### Secondary outcome analyses

Outcomes were also analyzed secondarily to identify any gender differences. Outcomes for male participants showed two significant time-by-group effects in the per-protocol analysis. Male participants in the Promillekoll group with reported app use increased their drinking frequency from baseline to follow-up, in comparison to control group participants (Z = 3.48, p = 0.001). One covariate was significant: number of app-use occasions [Z = 5.80, p < 0.001].

## Discussion

This study compared the effects of two smartphone apps for reducing overconsumption of alcohol at single-party occasions among Swedish university students to assessment-only controls. Both apps relied on mathematical estimates of blood alcohol concentration. The apps differed in content. The Promillekoll app offered information texts on different eBAC levels as well as strategies for avoiding risky drinking. The PartyPlanner app did not offer any explanatory texts but included a component allowing participants to plan their drinking in advance. Because of these differences in the apps, as well as difficulties in assessing which factors might be instrumental in any observed change in outcome, we chose to compare each app only to the control group and not to each other. Also, because the proportion of participants who actually used the app was significantly lower in the PartyPlanner group, we chose to focus on per-protocol analyses only, including participants who reported having used each app. We chose to control our analyses with three covariates. One concerned prior access to the Promillekoll app, which had been available for public access for over 5 months at the beginning of our study. Over one-third of app participants had used it before our study. The PartyPlanner was launched at the same time as this study and is not publicly available at this writing. The second covariate concerned access to other modes of help for alcohol consumption during the 12 months preceding the follow-up. We assessed three categories of help: medication, speaking to someone about alcohol consumption, and accessing other types of help such as internet-based interventions. About one in five participants reported having spoken to someone about their consumption in the previous 12 months. The third covariate concerned the number of times intervention group participants had used the app. The results showed only one time-by-group interaction, where Promillekoll participants showed a significant increase in drinking occasion frequency in comparison to controls. The proportion of students drinking more than the weekly Swedish recommendation of nine drinks for women and 14 for men appeared to remain stable over time at approximately 25–30 percent of all study participants.

It is not clear why Promillekoll users increased the frequency of their drinking at the same time that they did not, as a group, consume larger quantities of alcohol. We can only speculate that app users may have felt more confident that they could rely on the app to reduce negative effects of drinking and therefore felt able to drink more often.

The secondary gender-focused analyses suggest that male participants were the source of the increase in drinking frequency in the Promillekoll group. This finding suggests that it might be interesting to test gender-related hypotheses about the mechanisms steering the drinking behavior of male and female university students when using smartphone apps. One speculation is that real-time use of a smartphone app might trigger men to compete with their peers in a competitive “drinking game”. However, Promillekoll does not display eBACs over 0.08 percent, thus effectively setting an upper limit to how far the “game” can go. Interestingly, we did not see the same phenomenon in the PartyPlanner group. One reason could well be that attrition was significantly higher in the PartyPlanner group than in the other two groups, and where a higher proportion of men in the PartyPlanner group had dropped out. Moreover, these dropouts had higher levels of baseline alcohol consumption, a pattern not seen in the other two groups. It is also possible that individuals who might have been triggered to drink more frequently when having access to a smartphone app were present to a higher extent in the Promillekoll group.

Regarding the significance of covariates, the number of app-use occasions was associated with increases in outcomes in the analyses, possibly because those who drank more frequently had more opportunities to use the app than those who did not. This explanation could also apply to the other covariates, where an individual with higher levels of alcohol consumption might be more likely to try out apps for controlling it. They might also be more likely to speak to someone about their consumption.

The study took place over Easter and Walpurgis Night, both high-consumption holidays. These events may have led to seasonal spikes in consumption and may have affected the reported levels of consumption; however, this effect is most probably equal for all participants.

### Strengths and limitations

An important strength of this study is that it is, to our knowledge, one of the first effectiveness studies on apps for health-related behavior change for reducing risky alcohol consumption. A second strength is that this is the first randomized controlled study conducted with the Promillekoll app, which was released publicly by Systembolaget in the fall of 2012 following research-based development and a qualitative usability study. Thirdly, we studied university students, a highly important target group in that both risky alcohol drinking and smartphone app use are quite prevalent. It is possible to reach a large population of students easily and directly via e-mail addresses, facilitating this study and future research with this group. The design also ensures a minimum of human interaction, a possible advantage given the stigma attached to overconsumption of alcohol [43], and which potential participants might experience as an obstacle. The attrition rates in this study (over 30% overall; see Table 2) were somewhat lower than in other studies of electronically delivered SBIs, where attrition rates over time periods of 4–6 weeks ranged between 36.7 percent and 44.8 percent [44-46].

This study was also subject to several limitations. First, we had no information on possible differences between student union members who entered the study and those who did not. Among those invited to participate, only 16.9 percent gave informed consent. One reason for this could be that a certain proportion of our e-mails did not reach the recipients due to spam e-mail filters or to changes in e-mail addresses. We are therefore unsure of how representative this sample is of the total student population. What is clear, however, is that prevalence rates of problematic drinking in this sample were consistent with earlier studies targeting university students in Sweden and the US [1,2]. Secondly, the two smartphone interventions differed significantly in graphic design and technical presentation. Moreover, the Promillekoll app had been publicly available for over 5 months before this study was launched. These circumstances made it difficult to conduct any meaningful comparison between the two apps, so we chose to compare each of them individually to the control group. Under ideal laboratory conditions, we would have added the planning and follow-up function that was part of PartyPlanner to the Promillekoll intervention, in order to facilitate participants’ awareness of the extra pedagogical functions in the PartyPlanner app. In this case, the apps were produced by different designers, confounding design and format with content. Thirdly, the attrition rates in the two intervention groups differed significantly. Some of the participants who completed the follow-up left written comments on the PartyPlanner app. Their comments indicated that they disliked the technical web-based app solution, which required continuous internet connection. This may have caused time lags in app response that may have further deterred participants from using the app, and may have contributed to attrition. Our attrition analysis showed that completers and noncompleters in both the control and PartyPlanner groups differed on baseline characteristics measuring alcohol consumption, whereas Promillekoll participants did not. We are not sure why the Promillekoll app had a more equal distribution of baseline characteristics between the attriters and completers, but Promillekoll was released following a rigorous design process, and this may have led to a more pleasant user experience, which in turn led to higher retention. The Promillekoll app has also been extensively advertised in Sweden and participants may have been more curious to participate in its testing. The difference in attrition rates may also have contributed to our finding of significant differences for Promillekoll participants, since this sample may have been more representative in that men with higher levels of alcohol use—and possibly prone to engaging in competitive games—were retained in the sample. High-alcohol consumers in the PartyPlanner group, on the other hand, dropped out to a larger extent; these may also have been individuals with higher impulsivity, lower acceptance of frustration, and consequently, a lower tolerance for lag times. The results may have been tipped in favor of PartyPlanner, given that fewer of the individuals with higher levels of alcohol consumption participated in follow-up.

A final limitation in the study is the lack of objective user data available for the apps. For the Promillekoll app, this was due to the fact that the app was programmed as a standalone app without data transmission on usage to any server. Our research group had no influence on the design and programming of this app. The PartyPlanner app, on the other hand, was developed by the research group and the possibility of accessing objective user data was included in the design. However, technical difficulties complicated the extraction of these data. We chose instead to rely on self-reported user data on whether participants had used the app, and on how many occasions. While these data may be subject to error, particularly given the unclear definition of “app use” as well as the number of app-use occasions, the extent of the error is approximately comparable. Had we used objective data for one app and self-report data for the other, we might have compromised the reliability of our comparisons to the control group even more.

A final ethical issue of note in this study is the fact that controls were not offered any specific intervention to address their risky alcohol use. Control group participants and intervention group participants received a recommendation to contact student health services both at registration and at follow-up if they felt concerned about their drinking. Also, at the end of the study, a considerable proportion of the students were above the levels for recommended weekly consumption, but they were not offered any further interventions or referral.

## Conclusions

Overall, participation in our study did not seem to affect drinking in any of the three study groups. However, the Promillekoll app seemed to be associated with a negative effect in the form of an increased number of drinking occasions over one week. Our conclusion from this study is that eBAC calculation in the app form is not effective for reducing alcohol consumption among university students. Future development of apps with this purpose may require additional input to supplement eBAC feedback.

Our secondary analysis suggests that there might be gender differences in how apps are used in the context of risky drinking among university students. The Promillekoll app produced by the Systembolaget had one possible negative effect for the men, but not for the women. The PartyPlanner app, with the additional functionality of planning ahead and comparing real drinking events with plans, did not seem to negatively affect men. However, participants in this trial arm had a higher dropout rate, consisting to a larger extent of male participants with higher alcohol consumption, in comparison to the Promillekoll and control groups. Further research is thus necessary to explore gender differences in the use of apps in this context. Such research should investigate which app features are associated with higher participant retention, as well as whether app design needs to take gender factors into account.

Future research should use a uniform design for apps with different intervention components in order to control for the confounding effects of differing designs. This would enable isolation of component effects from design and technical differences between apps. Finally, an important area of future research is offering further help to individuals drinking more than the recommended weekly levels who are identified through such research. Although we referred all individuals concerned about their drinking to student health services in this study, we suspect that many might be reluctant to approach such services. Some might not be aware of the harmful nature of their drinking, whereas others might experience approaching student services as stigmatizing. One possibility would be to offer more indepth automated interventions to individuals drinking more than the recommended levels in a separate, secondary study.

## Competing interests

The authors declare that they have no competing interests.

## Authors’ contributions

MG, CA, AHB, and KS designed the study. MG carried out the data collection. MG, IR, and AHB were responsible for the statistical analyses. MG, AHB, CA, and KS drafted the manuscript. The PartyPlanner app was conceived by CA, MG, and AHB. All authors were involved in preparation of the final manuscript. All authors read and approved the final manuscript.
